# Lidocaine for Sodium Channel Toxicity in Diphenhydramine Overdose: Case Report

**DOI:** 10.5811/cpcem.41491

**Published:** 2025-04-30

**Authors:** Kassem Makki, David Mandil, Roger Hopson, Maxim Kashin, Roger Rothenberg, Noah Reisman, Brenna Farmer

**Affiliations:** * New York Presbyterian-Brooklyn Methodist Hospital, Division of Emergency Medicine; † New York Presbyterian-Brooklyn Methodist Hospital, Division of Internal Medicine, Pulmonary and Critical Care; ‡ New York University Langone Health, Division of Toxicology

**Keywords:** diphenhydramine overdose, sodium channel toxicity, lidocaine, case report, sodium bicarbonate resistance

## Abstract

**Introduction:**

Diphenhydramine overdose is a growing concern, particularly among adolescents influenced by online challenges. Traditionally managed with supportive care and sodium bicarbonate, severe cases may exhibit refractory symptoms due to sodium channel toxicity, necessitating alternative treatments.

**Case Report:**

A 28-year-old male with a history of anxiety and depression presented to the emergency department unresponsive, next to an empty bottle of diphenhydramine and wine bottles. Vital signs indicated hypotension and hypoxia. The patient was intubated and administered vasopressors. Initial electrocardiogram (ECG) showed a widened QRS complex and terminal R wave in lead aVR, suggesting sodium channel blockade. Treatment with multiple boluses of sodium bicarbonate was ineffective. Lidocaine (95 milligrams intravenously) was administered, resulting in improved ECG findings and patient stabilization. Subsequent care focused on supportive measures and treatment for aspiration pneumonia. The patient was extubated on day two and discharged on day seven to a behavioral health facility.

**Conclusion:**

This case underscores the effectiveness of lidocaine as a secondary treatment for diphenhydramine-induced sodium channel toxicity when standard sodium bicarbonate therapy fails. Lidocaine’s ability to restore myocardial conduction illustrates its potential as a critical intervention in toxicological emergencies.

## INTRODUCTION

Diphenhydramine is a commonly misused over-the-counter drug intended for allergies or as a sleep aid, with overdose rates particularly high among children and adolescents due to rising social media challenges like the TikTok “Benadryl Challenge” became popular.^1^ Among overdose deaths, diphenhydramine is the most common antihistamine found.^2^ Although perceived as an H1 receptor antagonist, it functions as an inverse agonist, affecting histamine and acetylcholine receptor activity.^3^ In overdose, effects can range from mild sedation and tachycardia to coma, seizures, and cardiac dysrhythmias.^4^ First-generation antihistamines like diphenhydramine cross the blood-brain barrier, leading to central nervous system effects, unlike second-generation antihistamines, which are more selective for peripheral receptors. Most drugs from both generations are metabolized hepatically via the cytochrome P450 system, but their duration of action differs significantly.^5^

After oral intake at therapeutic doses, diphenhydramine concentration peaks in the bloodstream within two to three hours. After first-pass metabolism, 40–60% of an oral dose enters systemic circulation, with excretion predominantly via urine. The drug’s half-life ranges from 4–17 hours, increasing in duration with patient age.^4^,^6^ Toxicity may manifest as antimuscarinic effects such as tachycardia, blurred vision, dry mucous membranes, and urinary retention. Sodium channel blockade from tricyclic antidepressant (TCA) effects can lead to QRS widening and subsequent cardiovascular collapse from dysrhythmias. Pediatric fatalities have been reported at doses below 500 milligrams (mg), with seizures noted at 150 mg. For adults, the fatal dose is estimated at 20–40 mg per kilogram (kg). Overdose treatment is largely supportive. However, antidotal therapy with physostigmine to treat the antimuscarinic effects and treatment of sodium channel blockade with sodium bicarbonate to treat dysrhythmia may be necessary.^7^

## CASE REPORT

**Image 1:**
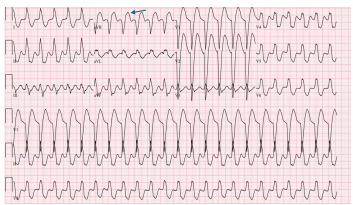
Initial electrocardiogram (8:52 am) notable for widened QRS to 162 milliseconds (blue bracket) with terminal R wave noted in AVR (blue arrow).

A 28-year-old man with a past medical history of anxiety, depression, stiff person syndrome, and Hashimoto thyroiditis was brought to the emergency department (ED) unresponsive after being found next to an empty bottle of diphenhydramine and multiple wine bottles. Upon presentation to the ED, vital signs were noted as follows: blood pressure, 104/27 millimeters mercury (mm Hg); heart rate, 90 beats per minute; respiratory rate, 18 breaths per minute; and oxygen saturation was low at 87% on room air. Due to the patient’s altered mental status, critical illness, and concern for airway protection, he was orotracheally intubated with etomidate and rocuronium and placed on mechanical ventilation. Post rapid sequence intubation sedation was done with propofol and dexmedetomidine. Due to the patient’s hypotension, norepinephrine was initiated. Initial electrocardiogram (ECG) demonstrated sinus rhythm at a rate of 54 beats per minute, a terminal R wave in lead aVR of 9 mm, and a QRS interval of 162 milliseconds (msec) (reference range: 80–100 msec), findings suggestive of sodium channel blockade like those seen in TCA overdoses^8^,^9^ (Image 1).

**CPC-EM Capsule**

What do we already know about this clinical entity?

*Diphenhydramine overdose can cause severe sodium channel blockade, leading to widened QRS, ventricular arrhythmias, and hemodynamic instability*.

What makes this presentation of disease reportable?

*Despite multiple sodium bicarbonate boluses, the patient had persistent QRS widening and ventricular tachycardia, which was successfully reversed with lidocaine*.

What is the major learning point?

*Lidocaine may serve as an effective alternative treatment for refractory sodium channel toxicity when sodium bicarbonate fails in diphenhydramine overdose*.

How might this improve emergency medicine practice?

*This case highlights the importance of recognizing lidocaine as a secondary therapy in diphenhydramine toxicity and optimizing treatment protocols for sodium channel blockade*.

**Image 2:**
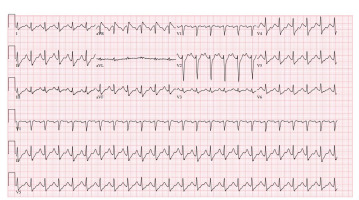
Electrocardiogram after sodium bicarbonate and amiodarone administration 10:12 am (notable for persistently widened QRS to 144 milliseconds with terminal R wave in AVR still present (blue arrow).

Poison control was called at this time; their recommendations were to give sodium bicarbonate and repeat vitals and ECGs on the patient. They did not specify dosing. They suggested that we look for co-ingestions as well, and if there was no improvement to possibly try extracorporeal membrane oxygenation. Venous blood gas (VBG) revealed severe metabolic acidosis with pH less than 7.00 (7.32–7.43), a markedly elevated partial pressure of carbon dioxide over 115 mmHg (40–60 mm Hg), and a lactic acid level exceeding 20 millimoles per liter (mmol/L) (0.5–2.0 mmol/L). To treat sodium channel blockade, the patient received 11 boluses of 8.4% sodium bicarbonate totaling 550 milliequivalents. A repeat ECG showed no improvement, with sinus tachycardia at a rate of 156, widened QRS complex of 174 msec, and a terminal R wave in lead aVR of 7 mm (Image 2). The patient’s blood pressure did improve to 122/24 after administration of norepinephrine post-intubation.

**Image 3:**
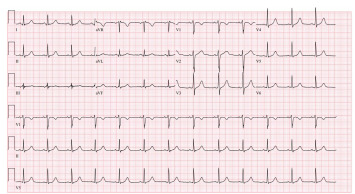
Electrocardiogram after lidocaine administration QRS duration decreased to 102 milliseconds 10:38 am – lidocaine administered at 10:30 am (per electronic health record).

Initially, this rhythm was interpreted as ventricular tachycardia ventricular tachycardia by the bedside team, who administered a bolus of 150 mg amiodarone and attempted synchronized cardioversion twice, with no appreciable response. Repeat VBG at this time demonstrated a pH of 7.31. Given the lack of appreciable ECG changes despite alkalinization, further sodium bicarbonate was not administered. The attending physician made the decision to administer 95 mg intravenous lidocaine based on the patient’s weight (1–1.5 mg/kg) at 10:16 am due to minimal improvement of V-tach and refractory V-tach. At 10:38 am the patient’s ECG improved, demonstrating sinus tachycardia with a rate of 140 beats per minute, QRS 104 msec, and a decreased terminal R wave amplitude in aVR to 2.5 mm (Image 3).

Activated charcoal, 100 grams, was then administered via nasogastric tube. The urine toxicology screen was negative for any commonly detected drugs of abuse. Serum acetaminophen and salicylate concentrations were undetectable. The patient was admitted to the intensive care unit (ICU), where he continued to receive supportive care and treatment for aspiration pneumonia. Given that sodium bicarbonate has considerable effects on their serum potassium concentration, the initial potassium once the patient was stabilized and then admitted to the ICU was 2.7 mmol/L. An initial potassium was not determined before the boluses of sodium bicarbonate were given. A serum diphenhydramine concentration obtained approximately eight hours after arrival was found to be elevated at 2,500 nanograms/mL. To note, the recommendations for diphenhydaramine dosing are 25–50 mg orally every 4–6 hours, with a maximum dose of 300 mg/day.

**Image 4:**
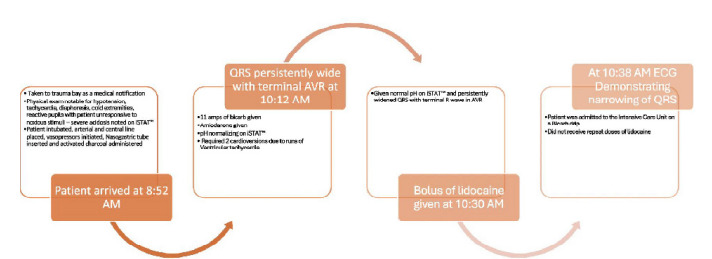
Electrocardiogram with normal sinus rhythm on day seven.

The patient was extubated on hospital day two and transferred to the general medical floor on hospital day three, during which time he admitted to psychiatry and hospital staff that the overdose was intentional. He was discharged to a behavioral health facility on hospital day seven where an additional ECG showed normal sinus rhythm (Image 4).

## DISCUSSION

This patient exhibited severe cardiovascular toxicity from diphenhydramine overdose as evidenced by his ECG changes. He presented with severe sodium channel toxicity secondary to diphenhydramine overdose, exhibiting hypotension, widened QRS, and acidosis. Despite initial treatment with sodium bicarbonate and amiodarone, the QRS complex remained persistently wide with a terminal R wave in aVR. After a bolus of lidocaine, the QRS narrowed significantly, and the patient stabilized. He was admitted to the ICU on a bicarbonate drip and did not require additional doses of lidocaine. A detailed timeline of this case can be seen in Figure below.

Diphenhydramine, an H1 histamine receptor inverse agonist commonly used for its antiallergic and sedative properties, works on many different pathways including the histamine and muscarinic receptors as well as sodium channels in the neurologic and cardiovascular systems. The ECG findings in this case demonstrated sodium channel blockade with a prolonged QRS interval (>100 msec) and terminal R wave in lead aVR, similar to ECG findings seen in TCA toxicity. The presence of a terminal R wave in lead aVR (terminal rightward axis deviation) and a widened QRS interval suggested severe toxicity, consistent with the patient’s clinical condition.^10^ These ECG changes were effectively managed with hypertonic sodium bicarbonate therapy in this scenario.

The literature provides multiple instances where these specific ECG alterations have been observed, such as the case of a 13-year-old female who overdosed on diphenhydramine. Treatment with sodium bicarbonate usually reverses these changes and improves symptoms.^11^ Unfortunately, although sodium bicarbonate improved our patient’s acidemia, it did not improve the cardiovascular toxicity as reflected by ECG.

The cardiotoxic effects attributed to diphenhydramine, including wide-complex tachycardia, arise from its blockade of fast sodium channels on cardiac membranes. This prolongs phase 0 of the cardiac action potential and delays electrical conduction through the heart, akin to the action of class Ia antiarrhythmics.^12^ Sodium bicarbonate counteracts the cardiotoxic effects by increasing the transcellular sodium concentration gradient, decreasing xenobiotic affinity for the sodium channel by increasing pH, and restoring normal myocardial conduction.^12^ In this case, a large amount of sodium bicarbonate was given with little, if any, improvement in the cardiac conduction abnormalities, despite effective alkalinization as demonstrated by VBG.

Lidocaine is a class Ib anti-dysrhythmic with affinity for inactivated sodium channels, resulting in decreased action potential duration and decreased effective refractory period.^13^ Lidocaine exhibits “fast on, fast off” sodium channel binding kinetics, meaning it rapidly binds to and inhibits the channel before quickly dissociating, restoring normal function. This property allows lidocaine to effectively compete with diphenhydramine, displacing it from the sodium channel. Despite its rapid dissociation, a single dose of lidocaine may lead to sustained improvement by transiently restoring normal sodium channel function, allowing endogenous recovery mechanisms—such as drug redistribution or metabolism—to reduce diphenhydramine’s inhibitory effects over time.^13^ This property allows lidocaine to “compete” with diphenhydramine and effectively displace it from the sodium channel. This case demonstrates the effective use of lidocaine in refractory sodium channel blockade unresponsive to sodium bicarbonate therapy and its utility for toxicologic Advanced Cardiovascular Life Support (ACLS). Lidocaine has become a second- or third-line antidysrhythmic in ACLS algorithms due to the evolution of the medical literature over the years.^13^

## CONCLUSION

This report highlights the successful use of lidocaine for refractory sodium channel toxicity following a diphenhydramine overdose in a 28-year-old male. Despite initial management with sodium bicarbonate, only lidocaine effectively reversed the severe cardiotoxic effects. This case emphasizes lidocaine’s potential utility in ACLS for toxicological emergencies. It also underscores the variability in treatment responses, urging further investigation into alternative therapeutic strategies for managing drug-induced cardiac disturbances.

## Figures and Tables

**Figure:**
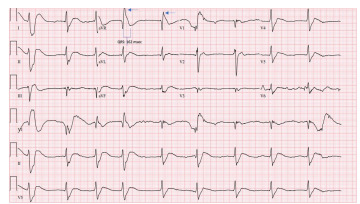
Case timeline. *bicarb*, bicarbonate; *i-STAT*, portable blood analysis system (Abbott Laboratories, Chicago, IL).
